# Impact of oligomerization on the allergenicity of allergens

**DOI:** 10.1186/s12948-022-00172-1

**Published:** 2022-04-29

**Authors:** Amin Moradi Hasan-Abad, Mohsen Mohammadi, Hamed Mirzaei, Mohsen Mehrabi, Hossein Motedayyen, Reza Arefnezhad

**Affiliations:** 1 Autoimmune Diseases Research Center, Shahid Beheshti Hospital, Kashan University of Medical Sciences, Kashan, Iran; 2 Persian Gulf Marine Biotechnology Research Center, The Persian Gulf Biomedical Sciences Research Institute, Bushehr University of Medical Sciences, Bushehr, Iran; 3 Research Center for Biochemistry and Nutrition in Metabolic Diseases, Institute for Basic Sciences, Kashan University of Medical Sciences, Kashan, Iran; 4 Department of Medical Nanotechnology, School of Medicine, Shahroud University of Medical Sciences, Shahroud, Iran; 5 Department of Anatomy, School of Medicine, Shiraz University of Medical Sciences, Shiraz, Iran

**Keywords:** Oligomerization, Allergenicity, Protein-protein interactions, Cross-linking, High-affinity IgE receptor

## Abstract

Type I hypersensitivity (allergic reaction) is an unsuitable or overreactive immune response to an allergen due to cross-link immunoglobulin E (IgE) antibodies bound to its high-affinity IgE receptors (FcεRIs) on effector cells. It is needless to say that at least two epitopes on allergens are required to the successful and effective cross-linking. There are some reports pointing to small proteins with only one IgE epitope could cross-link FcεRI-bound IgE through homo-oligomerization which provides two same IgE epitopes. Therefore, oligomerization of allergens plays an indisputable role in the allergenic feature and stability of allergens. In this regard, we review the signaling capacity of the B cell receptor (BCR) complex and cross-linking of FcεRI which results in the synthesis of allergen-specific IgE. This review also discusses the protein-protein interactions involved in the oligomerization of allergens and provide some explanations about the oligomerization of some well-known allergens, such as calcium-binding allergens, Alt a 1, Bet v 1, Der p 1, Per a3, and Fel d 1, along with the effects of their concentrations on dimerization.

## Introduction

Hypersensitivity reactions are generally classified into Type I, Type II, Type III, and Type IV. Type I hypersensitivity is an immediate reaction due to immunoglobulin E (IgE) antibodies against a soluble allergen [1]. It reveals itself in a range of troublesome to life-threatening diseases, from atopic eczema, seasonal hay fever, food allergies, drug allergies, asthma, allergic conjunctivitis, angioedema, urticaria (hives), eosinophilia, sweet itch to anaphylaxis [2]. These reactions are also known as atopic allergies. The term atopic is derived from the Greek word atopos, which means unusual or inappropriate. Allergic reactions begin with the exposure of the skin and mucosal surfaces to allergens resulting from IgE production, mast cell and basophil sensitisation and degranulation [2, 3]. The important impacts of the mediators (histamine, leukotrienes, and prostaglandins) are vasodilation and smooth-muscle contraction [4, 5]. Many people suffer from these disorders around the world, in a range of mild discomfort to rapid death. In general, the prevalence of atopic reactions is increasing and occurs in about 20 to 30% of the population [3, 4]. It is somewhat unclear why some people develop inflammatory reactions to these harmless substances. Multiple agents are responsible for susceptibility to allergic reactions, including environmental, hormonal and genetic factors [5].

FcεRI has a high affinity for the IgE antibody or allergen-IgE complex. It is reported that cross-linking of at least two FcεRI-bound IgEs by an allergen are needed to the degranulation of mast cell and basophils [6]. Previous studies have revealed that two major immunological properties of allergenic proteins play pivotal roles in inducing allergic reactions, including stimulation of B cell receptors (BCRs) to produce allergen-specific IgE antibody and capacity of the cross-linking of IgE bound to FcεRI on effector cells [6–8].

## The role of oligomerization of monovalent antigens in improving the signaling capacity of BCR

IgE synthesis in allergic reactions is induced following B cell activation through allergens binding to BCR [9]. The stimulation of this receptor results in the activation of Bruton’s tyrosine kinase (BTK), a key member of the BCR signaling pathway, leading to internalize BCR bound to allergens. B cells present allergen-derived peptides to CD4+ T helper2 (Th2) cells which play indispensable roles in B cell proliferation and its differentiation into IgE-secreting cells through releasing some cytokines and immune mediators.

Epitopes of a polyvalent antigen are able to cross-link at least two BCRs which in turn enhance B cell activation, proliferation, and differentiation into IgE-secreting cells [10]. However, monomeric soluble antigens may induce B cell unresponsiveness [11]. Recent studies have demonstrated that a monovalent antigen with a length of 17 amino acids is only able to activate B cells, while the oligomer form of short peptides could more effectively activate B cells [11–13]. It is revealed that the oligomer forms of peptides shorter than eight amino acids have the ability to activate B cells [11, 13]. Furthermore, the dimerizations of plant allergenic profilins rHev b 8 (rubber tree) and rZea m 12 (maize) considerably increases the IgE-mediated degranulation in rat basophilic leukemia cells [14]. These observations suggest that oligomerization of monomer forms of allergens more effectively enhances the signaling capacity of BCR through providing at least two identical epitopes and cross-linking of BCR [15].

## Oligomerization effects of monovalent antigens on enhancing cross-linking capacity of FcεRI on the effector cells

For the successful cross-linking of the FcεRI-bound IgE antibodies on the effector cells, allergens must have at least two IgE-binding sites (IgE epitopes) [16]. Furthermore, two different allergen-specific IgE antibodies with complementary paratopes are required for the degranulation of effector cells. These antibodies must bind to their receptors with an appropriate distance [17, 18].

Most of multivalent antigens are large molecules, but some allergenic proteins have low molecular weight. Monomeric allergens rarely have two identical epitopes due to their low molecular weight. Since the oligomer structure of an allergen can present a repetitive array of the identical IgE epitope, oligomerization of allergens displaying only one epitope is sufficient to cross-link FcεRI-bound IgE antibodies on effector cells and initiates an allergic reaction via the release of immune agents [7, 19, 20]. Moreover, the oligomer structure of allergens can cross-link BCRs and subsequently induce allergen-specific IgE synthesis more potent than their monomeric forms [7, 19, 21].

Although there are some reports pointing to the importance of the oligomerization phenomenon in allergenic properties of soluble antigens [7], the possible mechanism(s) involved in the dimerization of allergens has not been well identified yet. Therefore, we review protein-protein interactions, such as amino acid-base interactions and peptide bonds, resulting in the formation of the homo-oligomer structures in allergens. As mentioned above, these homo-oligomerization could increase the cross-linking of BCRs on IgE-B cells and FcεRI-bound IgE on other effector cells by providing at least two epitopes.

### Calcium-binding allergens

**Fig. 1:**
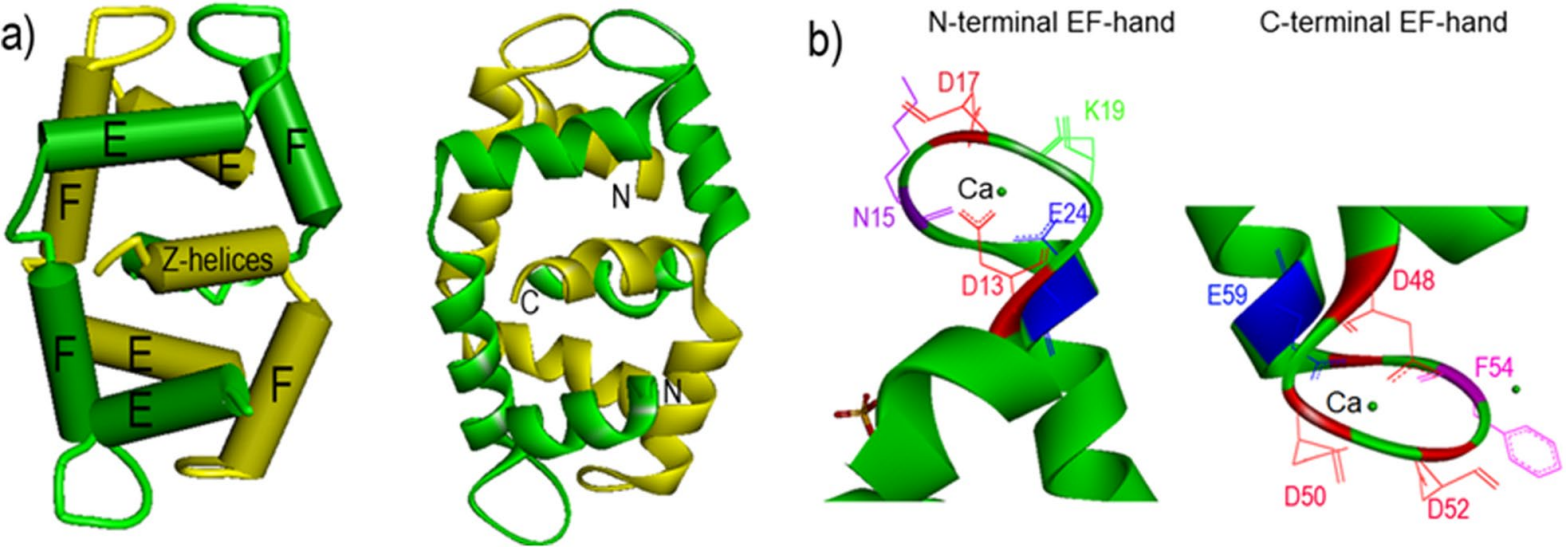
The ribbon model of intertwined Phl p 7 dimers. Monomers A and B are shown in yellow and green colors. **a** The N-terminal EF-hand calcium-binding motif of monomer A and C-terminal EF-hand calcium-binding motif of monomer B form upper EF-hands, while the C-terminal EF-hand calcium-binding motif of monomer A and N-terminal EF-hand calcium-binding motif of monomer B comprise lower EF-hands. The C-terminal Z-helices of two monomers form an intertwined equatorial belt. **b** This figure represents the side chains of the calcium-binding residues in the N- and C-terminal EF-hand calcium-binding motifs. The calcium ion (green) is in the middle of the loop. The Protein Data Bank (PDB) structures of Phl p 7 (PDB code: 1K9U) are shown by PyMol software

Some members of the calcium-binding protein family show important characteristics which can participate in allergic reactions in polysensitized individuals [21, 22]. Calcium-binding allergens, such as polcalcin, consist of two EF-hand calcium-binding motifs (helix-loop-helix domains) that connect to a hydrophobic helix (Z-helices) in C-terminal through a short linker (Fig. 1). To date, polcalcins from *timothy grass* (Phl p 7), *birch* (Bet v 4), and *common lambs quarters* (Che a 3) have been characterized and their structures in three dimension have been determined [21, 23, 24].

It is demonstrated that calcium-binding polcalcin possesses very high allergenic properties, while the calcium-depleted form of polcalcin (apo-polcalcin) fails to bind to IgE [22]. As shown in Fig. 1, monomeric polcalcin produces a dimer form according to a head-to-tail arrangement through the interactions between the helix-helix of EF-hand calcium-binding motifs, making a barrel shape with a hydrophobic cavity. This barrel is formed by calcium-binding domains in both the top and bottom of the barrel, and the E- and F-helices, which are located in upper and lower part of side, respectively [21].

Several studies have been performed on calcium-binding effects on the oligomerization of polcalcin [25, 26]. It is revealed that reconstruction of the dimer structure of polcalcin and its correct folding after thermal denaturation are largely related to the presence of calcium [21, 26]. The dimer form is the dominant structure of the calcium-binding polcalcin. Hypoallergenic polcalcin correlated with a mutation in a gene coded for calcium-binding sites is unable to make the dimer form. In addition to polcalcin, parvalbumin, as the main allergen of fish, is another allergen which could provide dimer forms through two EF-hand calcium-binding motifs [27–30].

### Alt a 1 allergen

Alt a 1 is a protein in the cell wall of *Alternaria* spores with unknown functions [14, 31]. It is known as the main allergen of *Alternaria alternata* fungus that induces an allergic reaction in approximately 90% of individuals suffering from *Alternaria* allergic [32–34]. In a study conducted by Chruszcz et al. on crystal structure of recombinant Alt a 1, it was indicated that the monomeric structure of Alt a 1 consists of a unique b-barrel form which can assemble to the dimer structure, a highly symmetric butterfly-like homodimer [14].

**Fig. 2:**
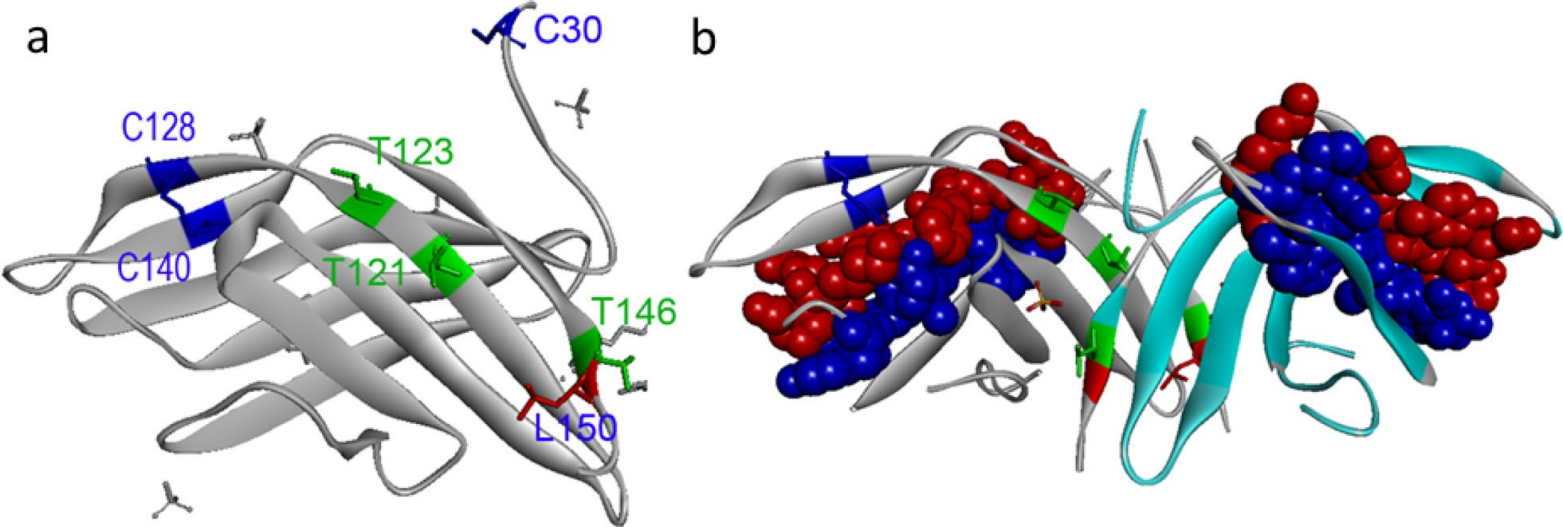
The ribbon representation of the Alt a 1 dimer. **a** Disulfide and hydrogen bonds that contribute to the dimeric form of Alt a 1 are shown on the monomer (PDB code: 3v0r). Two intramolecular disulfide bridges (C128-C140 and C74-C89) participate in stabilization of the β-barrel in monomeric form of Alt a 1 (intramolecular disulfide bridges between C74 and C89 is not shown in Figure. 1A) (PDB code: 3v0r). An intermolecular disulfide bridge formed by C30 from each monomer plays fundamental role in the dimerization of Alt a 1. In addition, hydrogen bonds participate in the formation of Alt a 1 dimer, including Thr121-Thr123, Thr146-Leu150 (PDB code: 4AUD). **b** The surface exposed and internal regions of Alt a 1 dimer are revealed in blue and red colors, respectively

The natural form of Alt a 1 is a dimer protein with a molecular weight of 30 kDa, showing two bands of 16.4 and 15.3 kDa, under reducing conditions on sodium dodecyl sulfate polyacrylamide gel electrophoresis (SDS-PAGE) [35]. Five disulfide bridges are presented in the Alt a 1 dimer, four of them are intramolecular and stabilize the β-barrel in each monomer. The last disulfide bridge contributes to the formation of the Alt a 1 dimer, and N-terminal cysteine (C30) covalently links to the equivalent residue in each monomer. This disulfide bridge holds two dimers in a “butterfly-like” configuration. Disulfide bonds and the mixture of polar and hydrophobic interactions donate high-temperature stability to the Alt a 1 dimer (Fig. 2). The main IgE epitope of the Alt a 1 dimer locates on the surface with the spatial condition which can cross-link Fc**ε**RI-bound IgE [14, 36].

In addition to the disulfide bond, hydrophobic and polar interactions, hydrogen bonds (Thr121, Thr123, Thr146, and Leu150), and N- and C-terminal regions contribute to the stabilization of the dimer structure of Alt a 1 (Fig. 2) [14]. Previous studies have shown that Alt a 1 forms a tetrameric structure, which can be attached to the flavonoid quercetin [37]. The stability of tetrameric Alt a 1-quercetin complex is pH-dependent, as observed in the other quercetin-bearing proteins. Tetrameric Alt a 1-quercetin complex is secreted from *Alternaria* spore. It is constant even in the low acidic environment such as bronchial epithelium with a pH value of 6, whereas this complex is broken down at pH less than 5.5 [36, 38, 39]. In a study conducted in 2012, the three-dimensional structure of this allergen was determined using X-ray crystallography and reported that there are four IgE antibodies binding regions on each monomer of this protein, all of them are located on the surface of the protein. Among these, two peptides (K41-P50 and Y54-K63) are located on the β1 and β2 strands of each protein monomer which strongly bind to serum IgE antibodies from *Alternaria alternata* allergies patients. Although some evidence pointing to the oligomerization of Alt a 1, as a classic and dimeric structure, is an important prerequisite for its allergenicity [14, 19], others have revealed that dimerization of this allergen may participate in its allergenicity, but is not essential. As reported mentioned in previous studies [19, 40], many important allergens, such as cockroach allergen Bla g 2, are protein monomers.

### Bet v 1 protein

Bet v 1, a member of pathogenesis-related (PR) proteins, is a major allergen of *birch pollen* [41, 42]. This allergen is highly conserved in pollen and plant foods. It is one of the most common allergens responsible for cross-reactivity between aeroallergens and food allergens [43, 44]. Various studies have reported that the cross-linking capacity and allergenic properties of Bet v 1 are largely dependent on the dimerization of this allergen [7, 45]. Schöll et al. reported that the Bet v 1 dimer can induce skin reaction in the skin prick test and activate specific B cells for IgE synthesis more effective than the monomeric form of this allergen [7]. Thus, the dimerization of Bet v 1 provides two IgE-binding sites that are required for the cross-linking of IgE on effector cells and BCRs on B lymphocytes.

**Fig. 3:**
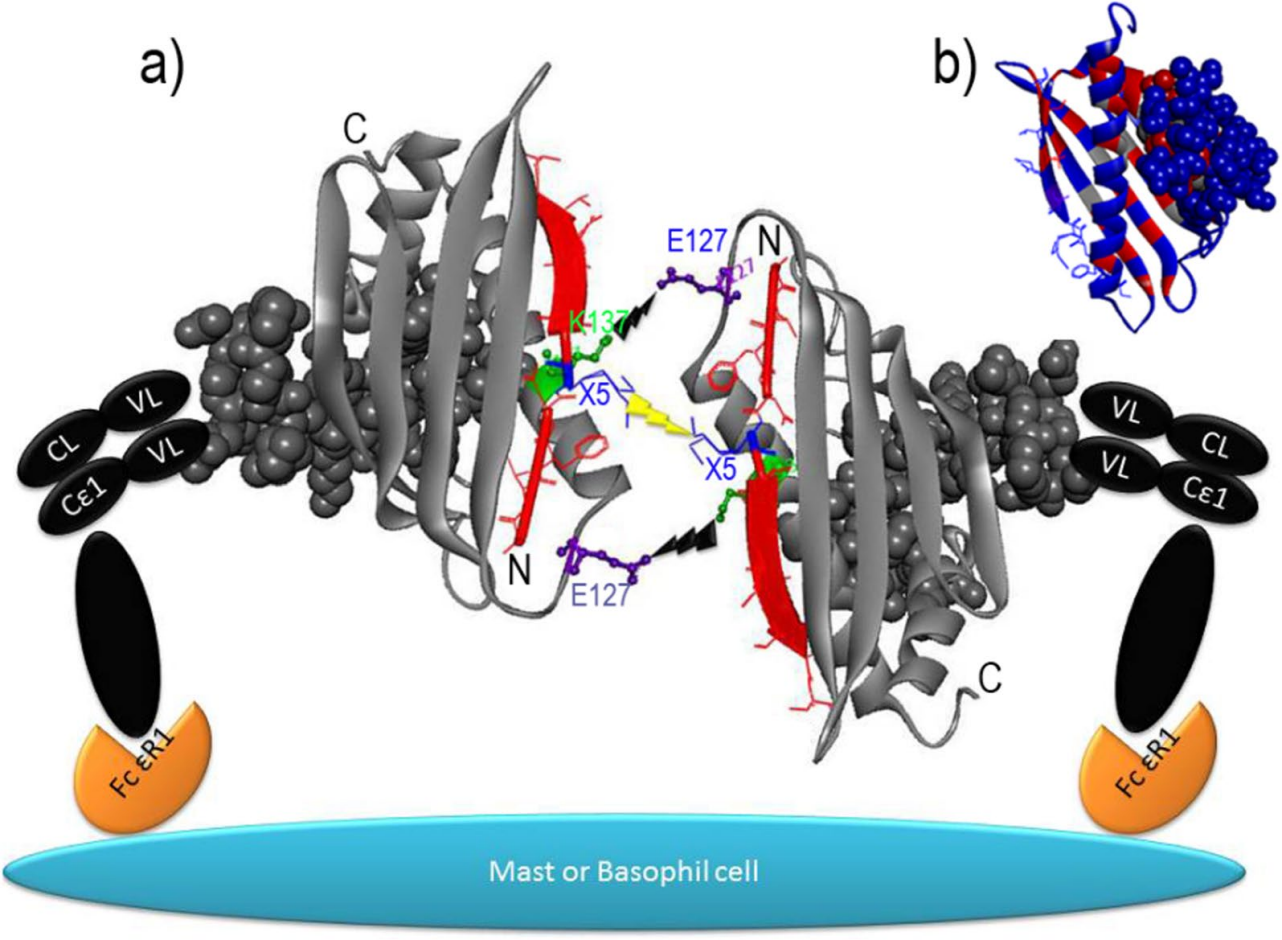
The ribbon representation of dimeric Bet v 1. **a** The salt bridge (between Glu 127 and Lys 137) and disulfide bonds (which is formed within cysteine 5) are indicated on dimeric Bet v 1. **b** This figure represents surfaces-accessible residues within the previously identified IgE epitopes in Bet v 1. The PDB structures of Bet v 1 (PDB code: 4BKD) are represented by PyMol software

The dimer form of Bet v 1 is generated by the interaction of two monomer forms of Bet v 1 through their N-terminal β-strands. This interaction is then strengthened by disulfide bonds and the salt bridge between Glu 127 and Lys 137 (Fig. 3). Kofler et al. found that the N-terminal region of the Bet v 1 protein, especially residue 5, plays an important role in the protein homodimerization. This finding is related to the intrinsic binding properties of the N-terminal β-strand in each monomer. In addition to its impact on the Bet v 1 homodimerization, several significant roles have been proposed for β-strand, such as IgE recognition, allergen uptake and presentation by primary dendritic cells accompanied by changes in cytokine profiles [45]. Previous studies show that four identified IgE epitope are located on the Bet v 1 dimer [45–48], which allows the cross-linking of monovalent FcεRI-bound IgE (Fig. 3).

### Der p 1 protein

**Fig. 4:**
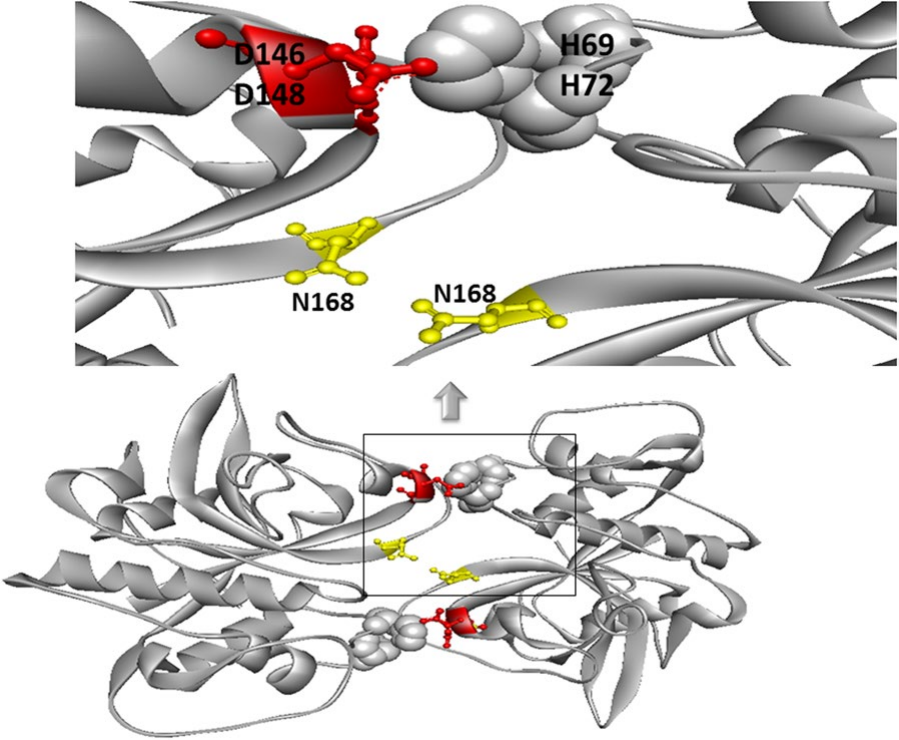
The ribbon diagram of the Der p 1 dimer. Spherical sign represents histidines 62 and 72 (H69 and H72). Aspartic acids 146 and 178 (D146 and 148), which interact with H69 and H72, are shown in red color, and asparagine 168 in the middle of the dimer interface is highlighted in yellow color. The PDB structures of Der p 1 (PDB code: 2AS8) are represented by PyMol software

Der p 1, a major allergen from *Dermatophagoides pteronyssinus*, is a 25-kDa glycoprotein, which approximately 70% of individuals with rhinitis, asthma, and dermatitis show an allergic reaction to this allergen [49, 50]. The crystallography studies have demonstrated that an extended interface, including three peptide segments (i.e., 69–75, 146–148, and 163–168) with polar and aromatic amino acids is constructed between each monomer and thereby leads to the dimer form of Der p 1. It is revealed that histidine 69 (His 69) and histidine 72 (His 72) have indispensable roles in the dimerization of the Der p 1. Having considered that disulfide bond, which participates in the dimerization of this allergen, is unstable in low acidic conditions, the allergenic properties of Der p 1 are largely dependent on pH, monomeric form of Der p 1 present at acidic pH while dimeric form is most stable in higher pH [51, 52] (Fig. 4). Therefore, Der p 1 shows greater allergenic properties under neutral and alkaline conditions.

**Fig. 5:**
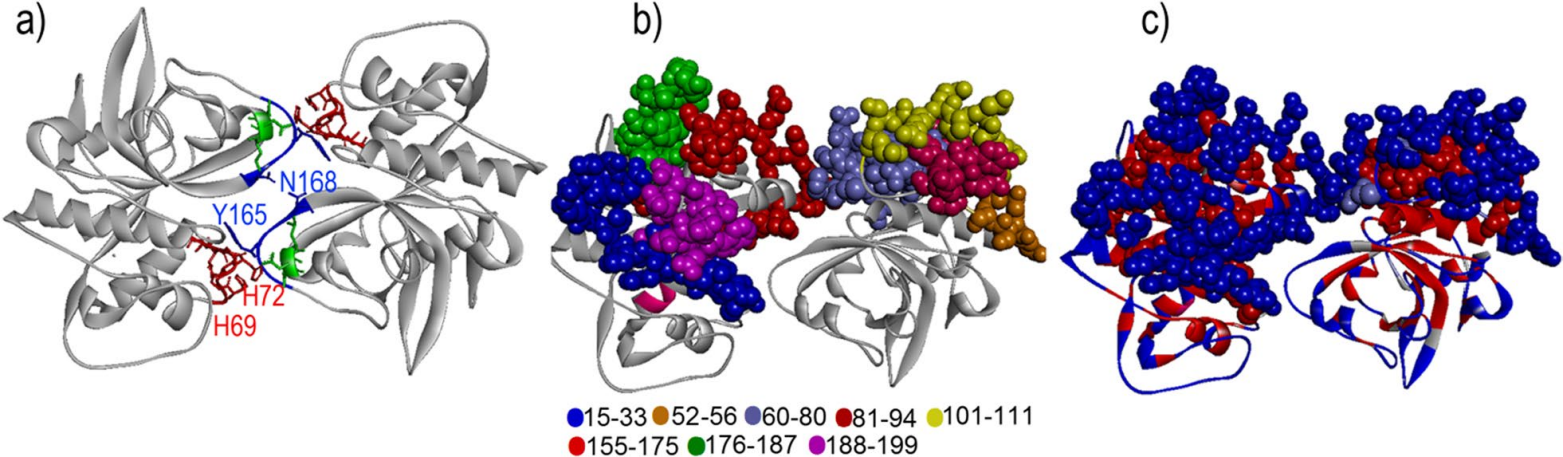
The position of the identified IgE epitope on the Der p 1 dimer. **a** This figure shows the Der p 1 dimer and residues (Asn 168, Tyr 165, His 69, and His 72), which contribute to the dimer formation of the Der p 1. **b** The surfaces-accessible residues within the previously identified IgE epitope was represented on dimeric Der p 1. **c** Figure reveals the surface exposed regions of allergen (Blue color)

In addition to the disulfide bond, other interactions contribute to the dimer formation and stability of the Der p 1 dimer. It is reported that a bifurcated hydrogen bond is formed by the interaction of the side chain of His 72 with Asp 146 and Asp 148 from another monomer (Fig. 4). Moreover, carbonyl oxygen hydrogen from His 72 interacts with the nitrogen of Tyr 165 from another monomer (Fig. 5). Asn 168 from each monomer interacts with another monomer in the center of interface and thereby participates in the dimerization of Der p 1 (Fig. 5).

Magnesium binds to Der p 1 via several residues, such as Asp 56, Glu 59, and Glu 91, which produces a site binding for magnesium. Furthermore, a hydrogen bond between the carbonyl oxygen of Leu 57 and magnesium ion has an important role in stabilizing magnesium binding to Der p 1 [52]. As shown in Fig. 5, the analysis of the surface accessibility of previously described IgE epitopes on the Der p 1 dimer revealed that peptide 15–33 is well accessible on the surface of Der p 1 and near to peptide 188–199 [53–55]. Therefore, these two peptides can create a discontinuous epitope (Fig. 5). Moreover, four well-accessible peptide segments, including 52–56, 60–80, 81–94, and 101–111, make linear or continuous epitopes. Peptide 155–175 is relatively buried within the dimer interface but can form a discontinuous epitope with peptide 176–187 or 60–80 (Fig. 5) [52].

### Per a 3 allergen

**Fig. 6:**
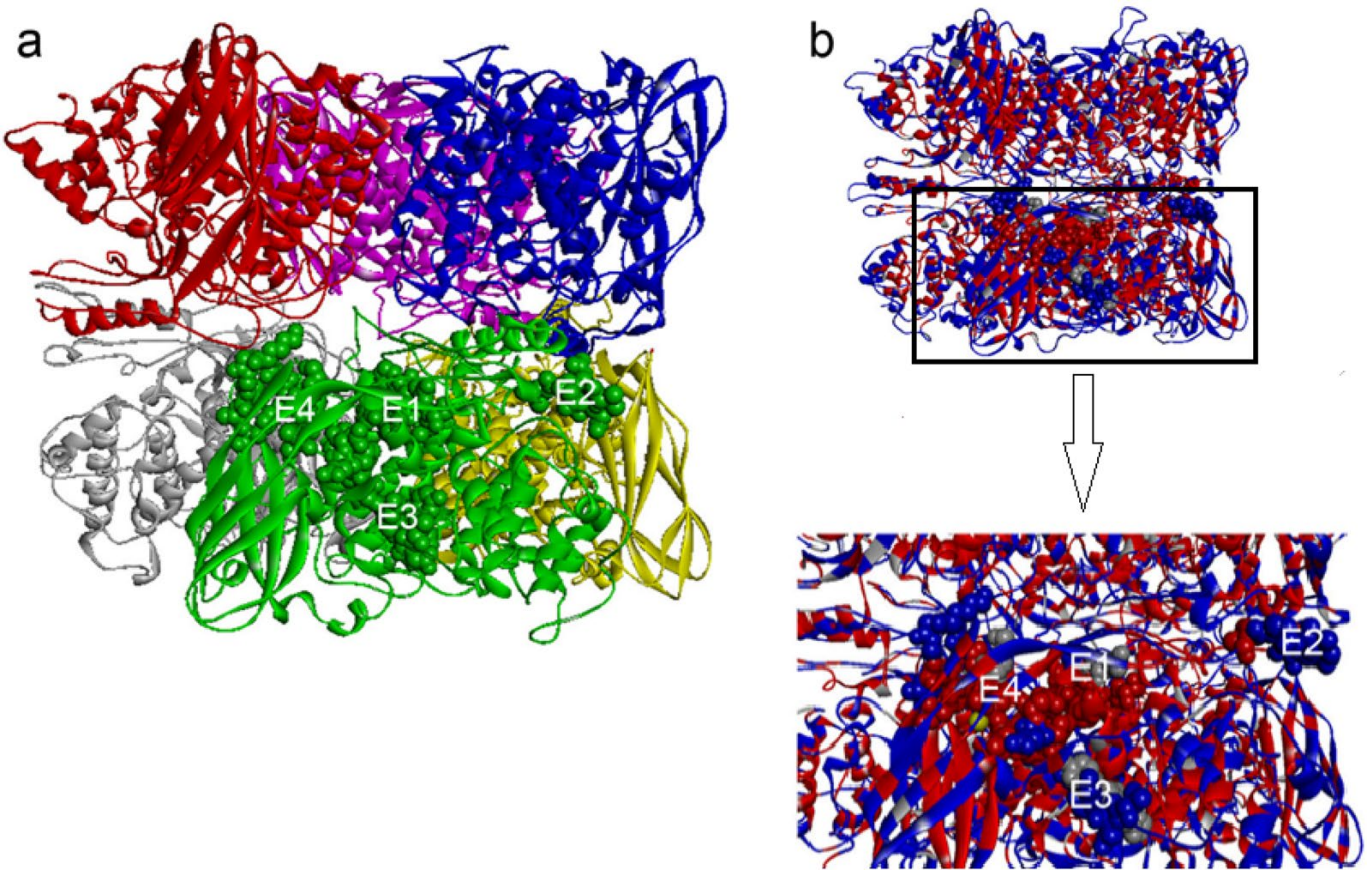
The ribbon representation of the Per a 3 oligomer. **a** Six monomers of Per a 3 make a hexameric structure. Monomers are shown in different colors. Four linear IgE epitopes, including E1 (400TVLRDPVFYQ409), E2 (466NNVDQI471), E3 (580VDKGHNYCGYPENLLI595) and E4 (595IPKGKKGGQAY605), have been identified on each monomer of Per a 3. These epitopes are highlighted in green color and their locations on the Per a 3 oligomer are mapped. **b** The surface exposed and internal regions of Per a 3 oligomer are indicated in blue and red colors, respectively

Per a 3, a hemocyanin in the hemolymph of cockroaches, possesses high identity in the amino acid level with storage proteins (hexamerins and arylphorins from the hemolymph in insects and arthropods). Per a 3 is identified as a major allergen from the American cockroach, which was recognized by the serum IgE from cockroach-allergic patients [56]. Mindykowsk et al. showed that Per a 3 makes a hexameric form similar to other hemocyanins. Mapping of IgE epitopes on a hexameric three-dimensional model revealed that all reported IgE epitopes located on the surface of the hexamer are freely accessible to Per a 3-specific IgE (Fig. 6) [57, 58]. The hexameric form of Per a 3 has highly thermos-stable properties, which allows it to remain for a long time as a high allergenic compound in house dust [56].

### Fel d 1 allergen

**Fig. 7:**
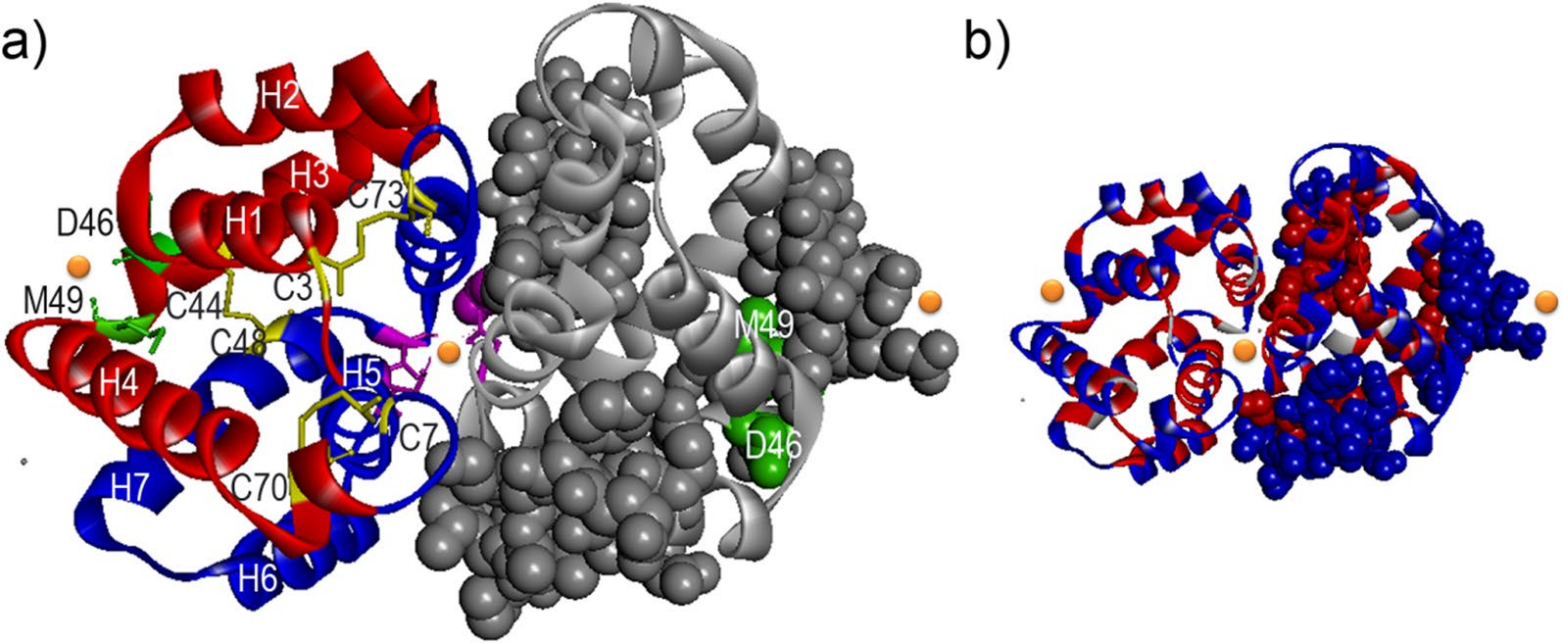
The ribbon representation of the Fel d 1 oligomer. **a** Chains 1 and 2 are shown in blue and red colors, respectively. Each chain contains four helixes (H) linked to each other by three disulfide bonds (C3–C73, C44–C48, and C7–C70). **b** The surface-accessible residues within the previously identified IgE epitope are shown on oligomer Fel d 1. Orange sphere reveals the calcium ion. The PDB structures of the Fel d 1 oligomer (PDB code: 2ejn) are indicated by PyMol software

Fel d 1, a 35-kDa tetrameric glycoprotein, has been reported as the main allergen in cat-allergic patients, which reacts with 90% of the serum IgE from these patients. Allergens from domestic cats cause approximately 10% of mild rhinitis but also life-threatening asthmatic reaction in the western world [59]. Heterodimer form of Fel d 1 is formed by two subunits, including chains 1 and 2 linked by three disulfide bonds among cysteine residues, such as Cys3, Cys73, Cys44, Cys48, Cys70, and Cys7 [59, 60]. The chains 1 and 2 consist of four helices, including H1 to H4 and H5 to H8, respectively. H5 and H8 helices participate in dimerization of Fel d 1 [60]. Tetrameric Fel d 1 made from two heterodimers (Fig. 7).

The interface generated between two monomer forms of Fel d 1 is constructed of a central hydrophobic core surrounded by a hydrogen bond made by charged residues. Three calcium-binding sites exist in tetrameric Fel d 1; one site is within the dimerization interface, and other sites are located on the side surface of each dimer in tetrameric Fel d 1. Three linear IgE epitopes were identified on the Fel d 1 allergen. These epitopes are located on the first and the end segments of H1–H2, H3–H4, and H5–H6, respectively [61].

## Effect of allergen concentrations on dimerization

In spite of amino acid-base interactions and peptide bonds, allergen concentration is a key factor in dimerization of monomeric allergens. Previous studies have shown that many allergens are able to make dimers or oligomer structures. The accumulations of monomeric antigens at higher concentrations provide a chance to increase their dimerization and thereby participate in enhancing protein immunogenicity [19, 62]. Although oligomerization and specific structural characteristics play indispensable roles in allergenicity of an allergen, these features are largely dependent on allergen concentrations. It is shown that allergens tend to have a monomeric form at low concentrations, while they shift to have dimer or oligomer forms at higher concentrations [57]. For example, the dimerization of the rBos d 2 is dependent on its concentration. The increasing concentration of this allergen progressively induces its dimer structure, resulting in a monomer-dimer equilibrium in the solution [19].

**Fig. 8:**
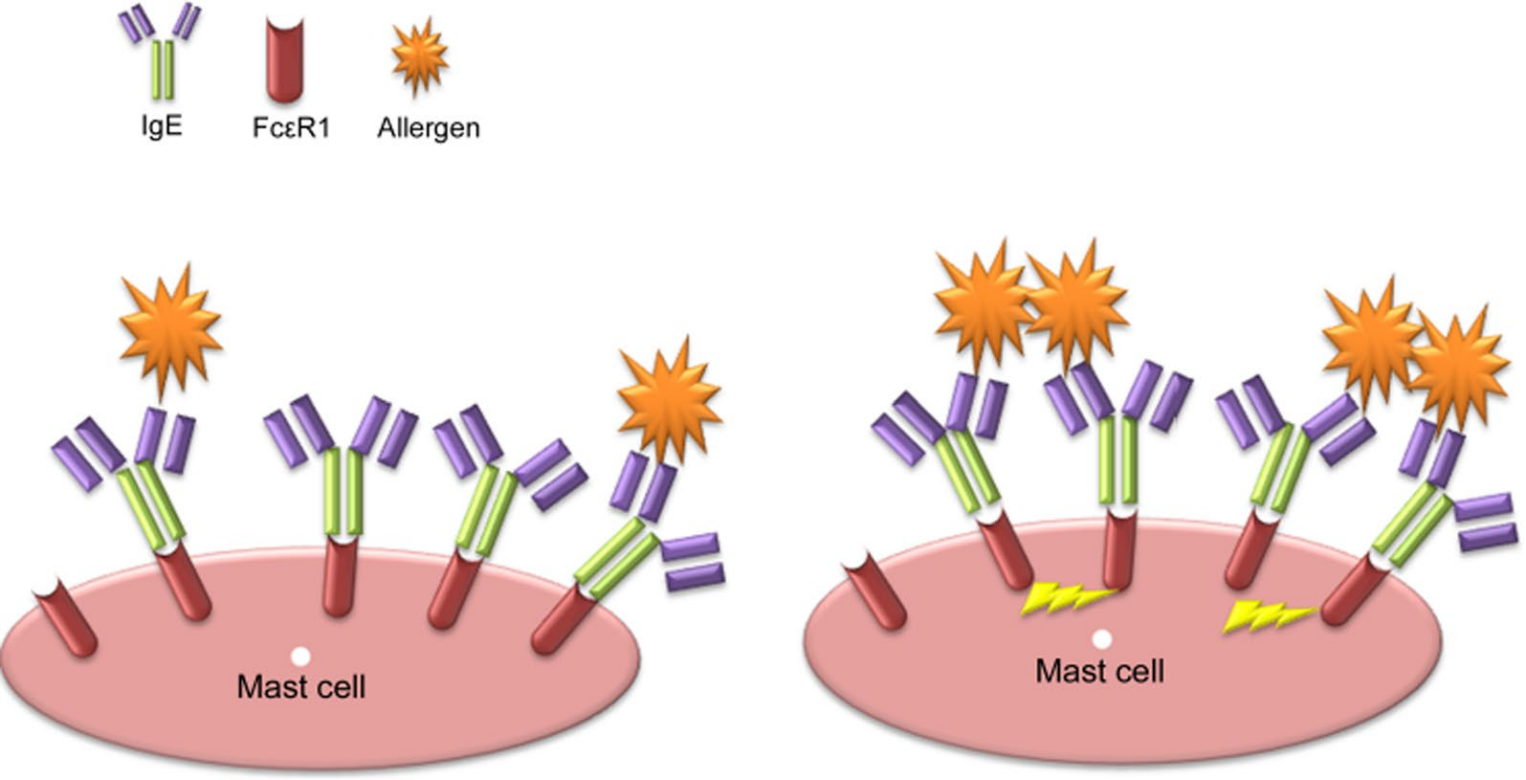
The dimer formation of the allergen on a mast cell. Individual allergens that present only one epitope, disable to cross-link the FcεRI-bound IgE antibodies (left). The increase of the local concentration of the allergen on a mast cell can increase the dimerization of allergens, leading to the cross-linking of FcεRI-bound IgE antibodies (right)

Rouvinen et al. demonstrated that approximately 80% of allergens are able to produce symmetric dimers or oligomers in crystals. Allergens frequently create a transient dimer structure at high concentrations; this observation is confirmed by the fact that their hypoallergenic variants have a monomer structure [19]. Kuriyan et al. reported that an increase in the colocalization of allergen molecules can significantly enhance their local concentrations [63]. In this regard, the local increase of monomer allergens bounding to the FcεRI-bound IgE antibodies enhance the interaction between allergen that leads to dimer formation and subsequently the cross-linking of FcεRI-bound IgE antibodies on the effector cells (Fig. 8) [19].

## Conclusions

Oligomerization of allergens play undeniable roles in the allergenic features and stability of allergens. In the present study, the protein–protein interactions were reviewed, which are responsible for allergen oligomerization (including amino acid-base interactions and peptide bonds). There are numerous studies pointing to at least two epitopes on the surface of allergens are necessary for the successful and effective cross-linking, however, some reports showing small proteins with only one IgE epitope (which form homo-oligomerization) can also cross-link FcεRI-bound IgE by providing two same IgE epitopes.

In conclusion, homo-oligomer structure in an allergen, which is mainly mediated by protein–protein interactions, has a key role in allergenicity of an allergen through providing at least two epitopes and enhancing the cross-linking of the BCRs on IgE-B cells and FcεRI-bound IgE on effector cells, but is not necessary. In addition, there are some reports revealing many important allergens are protein monomers.

## Data Availability

Not applicable.

## References

[1] Pritchard DI, Falcone FH, Mitchell PD (2021). The evolution of IgE-mediated type I hypersensitivity and its immunological value. Allergy.

[2] Sadati ZA, Motedayyen H, Sherkat R, Ostadi V, Eskandari N (2019). Comparison of the percentage of regulatory T cells and their p-STAT5 expression in allergic and non-allergic common variable immunodeficiency patients. Immunol Investig.

[3] Ghasemi A, Falak R, Mohammadi M, Maleki SJ, Assarezadegan M-A, Jafary M (2020). Incorporation of T-cell epitopes from tetanus and diphtheria toxoids into in-silico-designed hypoallergenic vaccine may enhance the protective immune response against allergens. Iran J Basic Med Sci.

[4] Moon TC, Befus AD, Kulka M (2014). Mast cell mediators: their differential release and the secretory pathways involved. Front Immunol.

[5] Gould HJ, Sutton BJ (2008). IgE in allergy and asthma today. Nat Rev Immunol.

[6] Gieras A, Linhart B, Roux KH, Dutta M, Khodoun M, Zafred D (2016). IgE epitope proximity determines immune complex shape and effector cell activation capacity. J Allergy Clin Immunol.

[7] Schöll I, Kalkura N, Shedziankova Y, Bergmann A, Verdino P, Knittelfelder R (2005). Dimerization of the major birch pollen allergen Bet v 1 is important for its in vivo IgE-cross-linking potential in mice. J Immunol.

[8] Knol EF (2006). Requirements for effective IgE cross-linking on mast cells and basophils. Mol Nutr Food Res.

[9] Rosenberg AS (2006). Effects of protein aggregates: an immunologic perspective. AAPS J.

[10] Pierce SK (2002). Lipid rafts and B-cell activation. Nat Rev Immunol.

[11] Avalos AM, Bilate AM, Witte MD, Tai AK, He J, Frushicheva MP (2014). Monovalent engagement of the BCR activates ovalbumin-specific transnuclear B cells. J Exp Med.

[12] Yang J, Reth M (2010). Oligomeric organization of the B-cell antigen receptor on resting cells. Nature.

[13] Avalos AM, Ploegh H (2014). Early BCR events and antigen capture, processing, and loading on MHC class II on B cells. Front Immunol.

[14] Chruszcz M, Chapman MD, Osinski T, Solberg R, Demas M, Porebski PJ (2012). *Alternaria alternata* allergen Alt a 1: a unique β-barrel protein dimer found exclusively in fungi. J Allergy Clin Immunol.

[15] Pali-Schöll I, Jensen-Jarolim E (2016). The concept of allergen-associated molecular patterns (AAMP). Curr Opin Immunol.

[16] Kanner BI, Metzger H (1983). Crosslinking of the receptors for immunoglobulin E depolarizes the plasma membrane of rat basophilic leukemia cells. Proc Natl Acad Sci.

[17] Zheng Y, Shopes B, Holowka D, Baird B (1991). Conformations of IgE bound to its receptor Fc. epsilon. RI and in solution. Biochemistry.

[18] Zheng Y, Shopes B, Holowka D, Baird B (1992). Dynamic conformations compared for IgE and IgG1 in solution and bound to receptors. Biochemistry.

[19] Rouvinen J, Jänis J, Laukkanen M-L, Jylhä S, Niemi M, Päivinen T (2010). Transient dimers of allergens. PLoS ONE.

[20] Niemi MH, Rytkönen-Nissinen M, Miettinen I, Jänis J, Virtanen T, Rouvinen J (2015). Dimerization of lipocalin allergens. Sci Rep.

[21] Verdino P, Westritschnig K, Valenta R, Keller W (2002). The cross-reactive calcium‐binding pollen allergen, Phl p 7, reveals a novel dimer assembly. EMBO J.

[22] Valenta R, Hayek B, Seiberler S, Bugajska-Schretter A, Niederberger V, Twardosz A (1998). Calcium-binding allergens: from plants to man. Int Arch Allergy Immunol.

[23] Neudecker P, Nerkamp J, Eisenmann A, Nourse A, Lauber T, Schweimer K (2004). Solution structure, dynamics, and hydrodynamics of the calcium-bound cross-reactive birch pollen allergen Bet v 4 reveal a canonical monomeric two EF-hand assembly with a regulatory function. J Mol Biol.

[24] Moghaddam MV, Fallahpour M, Mohammadi M, Varaee FR, Mokhtarian K, Khoshmirsafa M (2019). Identification of polcalcin as a novel allergen of *Amaranthus retroflexus* pollen. Allergol Immunopathol.

[25] Verdino P, Westritschnig K, Valenta R, Keller W (2002). The cross-reactive calcium-binding pollen allergen, Phl p 7, reveals a novel dimer assembly. EMBO J.

[26] Niederberger V, Hayek B, Vrtala S, Laffer S, Twardosz A, Vangelista L (1999). Calcium-dependent immunoglobulin E recognition of the apo‐and calcium‐bound form of a cross‐reactive two EF‐hand timothy grass pollen allergen, Phl p 7. FASEB J.

[27] Das Dores S, Chopin C, Villaume C, Fleurence J, Guéant JL (2002). A new oligomeric parvalbumin allergen of Atlantic cod (Gad mI) encoded by a gene distinct from that of Gad cI. Allergy.

[28] Swoboda I, Bugajska-Schretter A, Linhart B, Verdino P, Keller W, Schulmeister U (2007). A recombinant hypoallergenic parvalbumin mutant for immunotherapy of IgE-mediated fish allergy. J Immunol.

[29] Mohamadi M, Falak R, Mokhtarian K, Khoramizadeh MR, Sadroddiny E, Kardar GA (2016). Identification and characterization of main allergic proteins in cooked wolf herring fish. Iran J Allergy Asthma Immunol.

[30] Mohammadi M, Mokhtarian K, Kardar GA, Farrokhi S, Sadroddiny E, Khorramizadeh MR (2017). Expression of recombinant parvalbumin from wolf-herring fish and determination of its IgE-binding capability. Food Agric Immunol.

[31] Twaroch TE, Arcalís E, Sterflinger K, Stöger E, Swoboda I, Valenta R (2012). Predominant localization of the major *Alternaria* allergen Alt a 1 in the cell wall of airborne spores. J Allergy Clin Immunol.

[32] Vailes L, Perzanowski M, Wheatley L, Platts-Mills T, Chapman M (2001). IgE and IgG antibody responses to recombinant Alt a 1 as a marker of sensitization to *Alternaria* in asthma and atopic dermatitis. Clin Exp Allergy.

[33] Asturias JA, Ibarrola I, Ferrer A, Andreu C, López-Pascual E, Quiralte J (2005). Diagnosis of *Alternaria alternata* sensitization with natural and recombinant Alt a 1 allergens. J Allergy Clin Immunol.

[34] Twaroch T, Focke M, Fleischmann K, Balic N, Lupinek C, Blatt K (2012). Carrier-bound Alt a 1 peptides without allergenic activity for vaccination against *Alternaria alternata* allergy. Clin Exp Allergy.

[35] Deards M, Montague A (1991). Purification and characterisation of a major allergen of *Alternaria alternata*. Mol Immunol.

[36] Garrido-Arandia M, Bretones J, Gómez-Casado C, Cubells N, Díaz-Perales A, Pacios LF (2016). Computational study of pH-dependent oligomerization and ligand binding in Alt a 1, a highly allergenic protein with a unique fold. J Comput Aided Mol Des.

[37] Garrido-Arandia M, Silva-Navas J, Ramírez-Castillejo C, Cubells-Baeza N, Gómez-Casado C, Barber D (2016). Characterisation of a flavonoid ligand of the fungal protein Alt a 1. Sci Rep.

[38] Cianci M, Folli C, Zonta F, Florio P, Berni R, Zanotti G (2015). Structural evidence for asymmetric ligand binding to transthyretin. Acta Crystallogr Sect D Biol Crystallogr.

[39] Pasquato N, Berni R, Folli C, Alfieri B, Cendron L, Zanotti G (2007). Acidic pH-induced conformational changes in amyloidogenic mutant transthyretin. J Mol Biol.

[40] Li M, Gustchina A, Alexandratos J, Wlodawer A, Wunschmann S, Kepley CL (2008). Crystal structure of a dimerized cockroach allergen Bla g 2 complexed with a monoclonal antibody. J Biol Chem.

[41] Radauer C, Lackner P, Breiteneder H (2008). The Bet v 1 fold: an ancient, versatile scaffold for binding of large, hydrophobic ligands. BMC Evol Biol.

[42] Ferreira F, Ebner C, Kramer B, Casari G, Briza P, Kungl AJ (1998). Modulation of IgE reactivity of allergens by site-directed mutagenesis: potential use of hypoallergenic variants for immunotherapy. FASEB J.

[43] von SeutterLoetzen C, Hoffmann T, Hartl MJ, Schweimer K, Schwab W, Rösch P (2014). Secret of the major birch pollen allergen Bet v 1: identification of the physiological ligand. Biochem J.

[44] De Amici M, Mosca M, Vignini M, Quaglini S, Moratti R (2003). Recombinant birch allergens (Bet v 1 and Bet v 2) and the oral allergy syndrome in patients allergic to birch pollen. Ann Allergy Asthma Immunol.

[45] Kofler S, Ackaert C, Samonig M, Asam C, Briza P, Horejs-Hoeck J (2014). Stabilization of the dimeric birch pollen allergen Bet v 1 impacts its immunological properties. J Biol Chem.

[46] Gieras A, Cejka P, Blatt K, Focke-Tejkl M, Linhart B, Flicker S (2011). Mapping of conformational IgE epitopes with peptide-specific monoclonal antibodies reveals simultaneous binding of different IgE antibodies to a surface patch on the major birch pollen allergen, Bet v 1. J Immunol.

[47] Hecker J, Diethers A, Schulz D, Sabri A, Plum M, Michel Y (2012). An IgE epitope of Bet v 1 and fagales PR10 proteins as defined by a human monoclonal IgE. Allergy.

[48] Spangfort MD, Mirza O, Ipsen H, Van Neerven RJ, Gajhede M, Larsen JN (2003). Dominating IgE-binding epitope of Bet v 1, the major allergen of birch pollen, characterized by X-ray crystallography and site-directed mutagenesis. J Immunol.

[49] Arlian LG, Platts-Mills TA (2001). The biology of dust mites and the remediation of mite allergens in allergic disease. J Allergy Clin Immunol.

[50] Platts-Mills TA, Chapman MD (1987). Dust mites: immunology, allergic disease, and environmental control. J Allergy Clin Immunol.

[51] Li Y, Xiao W, Xiao K, Berti L, Luo J, Tseng HP (2012). Well-defined, reversible boronate crosslinked nanocarriers for targeted drug delivery in response to acidic pH values and cis‐diols. Angew Chem.

[52] De Halleux S, Stura E, VanderElst L, Carlier V, Jacquemin M, Saint-Remy J-M (2006). Three-dimensional structure and IgE-binding properties of mature fully active Der p 1, a clinically relevant major allergen. J Allergy Clin Immunol.

[53] Jeannin P, Didierlaurent A, Gras-Masse H, Elass AA, Delneste Y, Cardot E (1992). Specific histamine release capacity of peptides selected from the modelized der PI protein, a major allergen of *Dermatophagoides pteronyssinus*. Mol Immunol.

[54] Greene W, Thomas W (1992). IgE binding structures of the major house dust mite allergen DER PI. Mol Immunol.

[55] Collins S, Ball G, Vonarx E, Hosking C, Shelton M, Hill D (1996). Absence of continuous epitopes in the house dust mite major allergens Der p I from *Dermatophagoides pteronyssinus* and Der f I from *Dermatophagoides farinae*. Clin Experimental Allergy.

[56] Mindykowski B, Jaenicke E, Tenzer S, Cirak S, Schweikardt T, Schild H (2010). Cockroach allergens Per a 3 are oligomers. Dev Comp Immunol.

[57] Wu C, Lee M, Tseng C (2003). IgE-binding epitopes of the American cockroach Per a 3 allergen. Allergy.

[58] Wu CH, Lee MF, Liao SC, Luo SF (1996). Sequencing analysis of cDNA clones encoding the American cockroach Cr-PI allergens: homology with insect hemolymph proteins. J Biol Chem.

[59] Kaiser L, Velickovic TC, Badia-Martinez D, Adedoyin J, Thunberg S, Hallén D (2007). Structural characterization of the tetrameric form of the major cat allergen Fel d 1. J Mol Biol.

[60] Kroll Kristensen A, Schou G, Roepstorff P. Determination of isoforms, N-linked glycan structure and disulfide bond linkages of the major cat allergen Fel d1 by a mass spectrometric approach. 1997. 10.1515/bchm.1997.378.8.899 9377487

[61] Tasaniyananda N, Tungtrongchitr A, Seesuay W, Sakolvaree Y, Indrawattana N, Chaicumpa W (2016). A novel IgE-binding epitope of cat major allergen, Fel d 1. Biochem Biophys Res Commun.

[62] Nooren IM, Thornton JM (2003). Structural characterisation and functional significance of transient protein–protein interactions. J Mol Biol.

[63] Kuriyan J, Eisenberg D (2007). The origin of protein interactions and allostery in colocalization. Nature.

